# Public Voice via Social Media: Role in Cooperative Governance during Public Health Emergency

**DOI:** 10.3390/ijerph17186840

**Published:** 2020-09-18

**Authors:** Yang Yang, Yingying Su

**Affiliations:** School of Management, Harbin Institute of Technology, Harbin 150001, China; yfield@hit.edu.cn

**Keywords:** public voice, public health emergency, social media, policy evolution, product innovation, cooperative governance

## Abstract

With the development of the Internet, social networking sites have empowered the public to directly express their views about social issues and hence contribute to social change. As a new type of voice behavior, public voice on social media has aroused wide concern among scholars. However, why public voice is expressed and how it influences social development and betterment in times of public health emergencies remains unstudied. A key point is whether governments can take effective countermeasures when faced with public health emergencies. In such situation, public voice is of great significance in the formulation and implementation of coping policies. This qualitive study uses China’s Health Code policy under COVID-19 to explore why the public performs voice behavior on social media and how this influences policy evolution and product innovation through cooperative governance. A stimulus-cognition-emotion-behavior model is established to explain public voice, indicating that it is influenced by cognitive processes and public emotions under policy stimulus. What is more, as a form of public participation in cooperative governance, public voice plays a significant role in promoting policy evolution and product innovation, and represents a useful form of cooperation with governments and enterprises to jointly maintain social stability under public health emergencies

## 1. Introduction

As a positive extra-role behavior, voice has attracted extensive interests from scholars and gained substantial attention in the organizational behavior literature [1,2,3]. Public voice is a new type of voice behavior that refers to the behavior of citizens who share opinions on social media to improve the social status quo or prevent harmful practices [4]. As a pro-social behavior, public voice is vital for advancement and betterment of society [4], and it is believed that public voice plays an important role in the cooperative governance of government and other organizations (e.g., enterprises, non-profit organizations) under a public health emergency. Considering that public participation in public administration and policy formulation is beneficial to government performance, governments attach much importance to the public’s role in policy-making, especially in the areas of environmental governance, public health, and sustainable development [5,6,7,8]. In the face of extraordinary development problems, such as economic recession, public opinion in policy-making is extremely important [9]. Thus, to ensure the timeliness and efficiency of policy in the case of public health emergencies, the value of public voice, along with technical support from enterprise, should not be underestimated. In the COVID-19 epidemic, many governments have begun to cooperate with high-tech enterprises to formulate epidemic prevention and control policies such as the Health Code in China, COVIDWISE in Virginia, and Corona-Warn in Germany [10,11,12,13]. Public voice on social media has effectively promoted the evolution of epidemic control policy and tracking applications developed by enterprises, making an outstanding contribution to social stability. Thus, it is necessary to study public voice in public health emergencies in relation to the implementation of government policies and the promotion of enterprises’ product innovation. Public voice is also of great significance in further realizing cooperative governance.

Voice behavior refers to the extra-role interpersonal communication behavior in which organizational members actively make constructive suggestions to the organization for the purpose of improving work or organization status quo [14]. Previous studies on voice behavior have mainly focused on employee voice and customer voice within organizations; public voice in a broader context has received little attention. The importance of employee voice and customer voice for the sustainable development of enterprises suggests that the role of public voice in social improvement should not be underestimated, and is worthy of in-depth discussion [4]. Given that public voice can have wide ranging influence in terms of social change, this research focuses on its effect on the evolution of policy implemented under public health emergencies. Public voice in public health emergencies has several important characteristics: First, the target of public voice is more extensive. The targets of employee voice and customer voice are employees inside the enterprise and customers who have cooperative relationships with the enterprise, respectively. They often offer advice to the enterprise as a single identity. However, for public voice, the target is the general public, who have dual identities as policy participants and enterprise customers. Second, under cooperative governance, multiple subjects participate in policy-making, so the targets and content of public voice are also diverse. For example, voice to a government may relate to the implementation of policy, while that to an enterprise may focus on product improvement. Third, the channels for public voice are more diverse. Employees mainly voice to supervisors face-to-face or make suggestions through the internal social networks of an enterprise, and most customer voice occurs through the virtual community created by the enterprise. As social networking sites provide a more convenient platform for people to voice their concerns and make their voices heard, the public can voice through a variety of social networking sites [4]. Finally, the effect of public voice is more significant. Public health emergencies prompt the public to respond to the policy more actively and provide timely feedback [15], which forces the governments and enterprises to absorb public opinion as soon as possible to improve policies and products. Overall, research on public voice behavior is still in its infancy. The factors driving public voice and the mechanism of its action on government policies and enterprise product innovation are unclear. The purpose of this study is to address this gap and further explore the role of public voice in promoting cooperative governance under public health emergencies.

The main contributions of this paper are threefold. First, it extends the literature on voice behavior. Most studies on voice behavior have focused on employee voice and customer voice. Under cooperative governance, the public is a participant in government policy as well as a customer of enterprises, yet the mechanisms for the influence of public voice on policy and product are not clear. This paper focuses on the dynamic role of public voice in policy-making and evolution and product innovation. Second, it constructs a dynamic model of public voice to promote policy implementation under public health emergency. Studies of public participation have mainly focused on its effect on environmental projects and decision, as public participation is seen as highly valuable and necessary to achieve the goal of environmental pollution control [8,16,17]. However, the voice behavior of the public in the formulation and evolution of policies in public health emergencies is unknown. Finally, this paper extends the literature on cooperative governance in a public health emergency and attaches more importance to the role of public voice in the process of collaborative.

This research uses China’s Health Code policy under COVID-19 as an example. This is an epidemic prevention policy whose implementation relies on a health rating system developed by Alibaba, Tencent, and other firms. The system uses opaque algorithms and individuals’ data, such as physical condition and contact with an infected person, to make judgments about the infection risk of system users [18]. The system then generates a QR code corresponding to this risk level that is used as a passport. Based on the evolution process of Health Code policy, this paper downloads comments about the Health Code policy to do research. This study uses the qualitative research method of grounded theory to explore the factors driving public voice and reveals the dynamic mechanism of its influence on policy formulation and product innovation. Further, this research provides support for cooperative governance involving government, enterprises, and the public under public health emergencies.

## 2. Literature Review 

### 2.1. Voice Behavior

The concept of voice was first proposed by Hirchman in the field of economics. It has been further developed in the field of organizational behavior [19]. Currently, voice behavior is divided into employee voice, customer voice, and public voice. Most research on voice behavior has been in the field of organizational behavior and mainly aimed to explain employee voice within organizations. Van dyne and Lepine define employee voice behavior as a positive extra-role behavior focused on improving existing working methods and procedures through constructive suggestions; they emphasize the ‘promoting’ role of employee voice for the organization [14]. Van dyne et al. further expand the concept, pointing out that voice includes not only suggestions for improvement, but also concerns about the organization [20]. On this basis, Liang et al. clearly divide voice behavior into promotive voice and prohibitive voice [21]. Promotive voice refers to innovative ideas or suggestions put forward by employees to improve the overall operation of the organization, while prohibitive voice refers to employees’ attention to work practices, and events and employee behaviors that are not conducive to the development of the organization [21]. Employee voice is widely considered a valuable and positive extra-role interpersonal communication behavior, a kind of organizational citizenship behavior that plays an important role in the team and organization. Scholars have conducted in-depth research on the influential factors and outcome variables of employee voice. Previous studies indicate that personal characteristics, leadership, and organizational climate can influence employee voice, which will be beneficial to organizational betterment [2,3,22,23,24,25,26,27,28,29]. Additionally, the approaches of employee voice are also optimized due to the development of the Internet [30].

With the aggravation of market competition, customer participation becomes crucial for the product and service innovation of enterprises, and enterprises have created brand virtual communities to gather customers’ ideas and opinions. Research on voice behavior has also expanded from the internal voice of the organization to the field of consumer behavior. On the connotation level, Griffin and Hauser regard customer requirements as customer voice, holding the view that customers would sort their needs according to importance and convey them to enterprises [31]. Enterprises can then develop new products based on customer requirements. Lee et al. expand the connotation of customer voice and define it as a description of customers’ needs and expectations or preferences and dislikes, including the pursuit of rights and interests, suggestions for new products and services, and complaints about previous use experience. Earlier definitions of customer voice are based on customer needs, but with advances in research on employee voice within organizations, scholars have begun to redefine customer voice from the perspective of role orientation. Ran and Zhou clearly define customer voice as the extra-role communication behavior in which customers actively make suggestions or express opinions to improve the status of enterprises; this kind of behavior belongs to the category of customer citizenship behavior [32]. At the dimension level, most previous studies on customer voice divide it into two categories: customer satisfaction and customer complaint [33]. With the deepening of research, scholars find that customers not only express dissatisfaction regarding product and service providers, but also express satisfaction and praise, and make their own suggestions. Therefore, with reference to the classification of employee voice by Liang et al., customer voice can be divided into promotive voice and prohibitive voice [21]. Promotive voice refers to the innovative ideas and suggestions of customers regarding improvements to the efficiency of enterprises, while prohibitive voice refers to the expression of opinions on actual and potential problems within the products, services, or management of an enterprise that are harmful to the enterprise or its customers. As the input behavior of customers to enterprises, customer voice can urge enterprises to innovate products and services to meet the needs of customers, thus improving customer satisfaction and maintaining customer loyalty. It can also help enterprises correct errors, provide solutions to problems, and improve enterprise performance [33,34].

With the rapid development of social media, people can express their views on social issues more directly and conveniently, and research on voice behavior has been further extended to a broader social life context. Public voice behavior refers to citizens sharing opinions on social media to improve their social status quo or prevent harmful practices. It is essentially a pro-social behavior [4]. Public voice channels have begun to focus on social media, because in the modern world, social media presents extensive information; people express their concern about education, security, the environment, work-life balance, and many other issues online. Moreover, the diversity and openness of social media provides a broad platform for public expression. The public can conduct online voice behavior through third-party social media and public participation is increasing. However, research on public voice based on social media is still in its infancy and is uncommon. Bhatti et al. explore the mechanism of the effect of individual moral identity and proactive personality on public promotive voice and prohibitive voice based on self-consistency theory [4].

Research on voice behavior as discussed above has several characteristics. First, the research field has shifted from intra-organization to a broader social background. Second, the voice subjects present a change trend of ‘employee-customer-public’. Third, the targets of voice behavior change from organizational practice to general social phenomena. Fourth, the form of voice presents the evolution trend of “face-to-face-virtual community-social media”.

### 2.2. Public Role in Public Policy

Citizen participation in the formulation and consultation of public policies is an important way to strengthen and support modern democracy [35]. Regarding the influence of public participation on policy, most research reveals extensive interest in environmental protection and pollution control, as public participation can help decision makers recognize public concern and demands, and handle environmental conflict in a more flexible manner [36,37]. Fu and Geng explore the influence of public participation and regulation compliance on ‘green development’ with panel data from 30 provinces in China from 2004 to 2014, finding that public participation can lead enterprises to improve compliance and thus promote green development [8]. Regional environmental quality (REQ) is a comprehensive indicator of emissions of waste gas, waste water, and waste solids, and its improvement requires coordination between governance and public participation. Public participation can be coordinated with governance to effectively improve REQ effectively, and further promote the optimization of environmental governance system [38]. The arrival of the Internet era has changed the method of public participation. As a branch of e-government, e-participation has been widely examined by scholars. Considering that public participation is a voluntary activity, whether the public is willing to participate is the decisive factor affecting the success of e-government platforms. Scholars consider that in addition to demographic differences, willingness to use an e-participation system is affected by system technical factors, personal incentive factors, and social capital factors. Based on the unified theory of acceptance and use of technology, planned behavior theory, social capital theory, and other information system theories, previous studies have explored the willingness of the public to use the e-community to participate in policy-making and provide strategic suggestions for governments to improve e-government platform [39,40]. 

### 2.3. Product Innovation

Product innovation is an important focus in the innovation research field and is key for enterprises to obtain sustainable competitive advantage. At present, there is no unified definition of product innovation in academia. Katila and Ahuja define it as change in design attributes—such as technology, appearance, quality, and structure—relative to the existing products of an enterprise. This is also known as technological innovation or design innovation [41]. The Organization for Economic Co-operation and Development defines product innovation as the process leading to a new or significantly improved product or service [42]. Various scholars’ definitions of product innovation, identify two aspects: entity product innovation and service-related innovation. According to the different degree of innovation, product innovation can be divided into radical innovation and incremental innovation [43]. Rapid change in the external environment drives enterprise innovation; enterprises can only achieve long-term development by constantly producing more competitive products according to the needs of users. As an external innovation resource, customer voice can be regarded as a gift given by users to enterprises to help them carry out product innovation based on the collective wisdom [44,45,46]. Customer voice provides valuable information for enterprises, which can help product designers and engineers to understand customers’ needs and preferences, and turn them into key objectives of product improvement by making targeted adjustments to products and services to meet the needs of users [47]. Further, customer voice can help enterprises identify the product attributes to which customers pay most attention and focus on product improvement and new product development [48].

### 2.4. Cooperative Governance

Governance refers to processes and structures in public decision making and may involve the participation of multiple agents, such as governments, corporations, and the public, with the aim of carrying out a public purpose that cannot be accomplished by single force [49]. Cooperative governance is not limited to formal government-initiated arrangements, but involves diverse kinds of multi-partner governance related to a wide range of fields [49]. For example, because of the production of pollution, enterprises take the greatest responsibility for environment contamination control. However, as it is difficult for governance goals to be achieved through the actions of a single enterprise, so governance among enterprises is indispensable [50]. With regard to cooperative governance among governments, Zhang et al. find that superior government should supervise heterogeneous local governments and increase penalties for non-cooperative parties to improve the efficiency of haze pollution control [51]. Further, cooperative governance can provide guidance for participatory governance by the public [4]. Studies of cooperative governance involving public participation have focused on environmental governance and sustainable development. When making local energy decisions, local governments should be given more autonomy and sufficient capacity to strengthen public participation. What is more, public opinion ought to be taken into consideration when developing policy [52]. Studies show that policy-making style presents convergence to the cooperation among government, public and non-profit organizations. As the government may lack the necessary resources to deal with issues, they rely on other subjects to provide support to ensure policy utility [53].

To summarize, there are several problems needing to be solved: First, research on public voice is not mature and more studies are needed to clarify its antecedents as well as its effects on policy implementation and social development. Second, it is undeniable that the public plays a crucial role in environmental governance, but the role of public voice behavior in policy-making and implementation under public health emergencies is still unclear. Third, the role played by public voice in cooperative governance and how this happens deserve exploration.

## 3. Data Analysis

### 3.1. Case Background

At the beginning of 2020, the outbreak of COVID-19 brought great impact on people’s life and work. In order to contain the spread of novel coronavirus and speed up the normalization of production and life, on 7 February 2020, Yuhang first launched the Yuhang Health Code. And on 11 February 2020, Hangzhou launched the Hangzhou Health Code to implement “green code, red code, yellow code” three-color code dynamic management [18]. The implementation of this policy has aroused widespread concern of the people all over the country, and local governments have followed up and implemented a local version of code in few weeks [18]. The implementation of Health Code policy is assisted by the QR rating health code system developed by Alibaba, Tencent, or other firms. When registering, individuals should provide their names, ID numbers, phone numbers, and answer a series questions about physical health conditions and travel trajectory to get the initial rating [54]. In addition, the rating changes according to individual real-time data, which consists of individuals’ travel history, directly related health information, overall medical test results, and overall risk assessment from individuals’ reports, information from GPS (Global Positioning System), telecommunications supplier, consumption record, QR code usage record, etc. The system assesses individual’s infection risk and generates green, yellow, or red codes according to individual’s data [55]. People with green codes have a very low probability to be infected and can move around freely, while people with yellow codes have a risk to be infected to some extent and should be quarantined for a week. People with red codes are at great risk of infection and need to be quarantined for 2 weeks. During the quarantine, if people with yellow or red codes check in on the app every day, the codes will turn green at the end of quarantine periods. And if the real-time information shows that people with green codes have gone to a high-risk area or been in contact with an infected person, the code will turn yellow or red as well [10]. Up to August 2020, the Tencent Health Code covers a population of 9 hundred million people, more than 400 cities and counties, and more than 5100 villages in China, with a cumulative total of 42 billion visits [56]. With the evolution of the Health Code policy, the effective circulation of personnel from all over the country has met the needs of residents’ normal life and enterprises’ resumption of work and production. At present, residents only need to provide a real-time QR code generated in a mini-app embedded in Alipay (Alibaba, Hangzhou, China) or WeChat (Tencent, Shenzhen, China) to the guard, they can move around [54]. In the Health Code policy implementation process, the high-tech enterprises not only provide technical support to develop the health code system, but also participate in the formulation of policy standards and establishment of policy platforms. For example, Alibaba and Tencent have been fully involved in the formulation of national standards for the personal Health Information Code series [57,58]. Besides, during this process, the public is actively voicing on the implementation and evolution of the Health Code policy as well as improvements of health code application on social media. In the official Weibo of People’s Daily, tweets about the Health Code policy get plenty of comments and followers, most of which are advice for policy implementation and system improvement. For example, the tweet about the Hangzhou Health Code has 7227 comments and 79,547 followers. The government press conference and reports about enterprises confirm the public voice does play an important role in the evolution and promotion of Health Code policy and the voice is fully considered and adopted by government and enterprises when making decisions. On the joint prevention and control conferences of COVID-19, the government spokespersons provided response to public concern and the governments also instructed local government and related enterprises to take measures to meet public voice. In addition, the enterprises responded to public voice as well. In the Government Affairs Strategy Conference, Yuepeng Qiu, vice President of Tencent, said that they had updated the system more than 50 times.

### 3.2. Data Collection and Analysis

This study adopts a dynamic research perspective, and takes the dynamic evolution of health codes policy as an example, focusing on exploring how public voice promoted the improvement of products by enterprises and the implementation of policies by the government under a public health emergency. The core of grounded theory emphasizes the process of collecting and analyzing original data. In the data collection stage, the researcher takes the evolution process of the Health Code policy as the time axis, and collects public comments under the official microblog of the People’s Daily as the research object. Data analysis included the following stages: Firstly, open coding is used to identify phenomena, define concepts, and discover categories from the original data. Secondly, axial coding is carried out to further analyze to get the main category. Thirdly, selective coding is used to find the core category, and systematically connect it with other categories to construct a logical relationship. In the whole coding process, researchers keep supplementing the material. Finally, the selective coding is analyzed and theoretical construction is carried out, and the density, variation, and high integration of theoretical concepts are adjusted to form a theoretical framework. The qualitative analysis software Nvivo 11.0 (QSR International, Melbourne, Australia) was used for the analysis of this study.

#### 3.2.1. Open Coding

Open coding is to analyze the original data word by word, so as to summarize the initial concepts and categories in the original data. Following the process of “tagging-conceptualization- categorization”, the researchers analyzed the collected data word by word and refined the semantics of the data to obtain the corresponding concepts and categories. Examples are shown in Table 1.

#### 3.2.2. Axial Coding

The purpose of axial coding is to explore the potential logical relationship between categories and develop main categories. This study classifies different categories according to their relationship at the conceptual level, and concludes eight main categories, which are divided into three classifications. The main categories and their corresponding classifications and relations are shown in Table 2.

#### 3.2.3. Selective Coding

**Figure 1:**
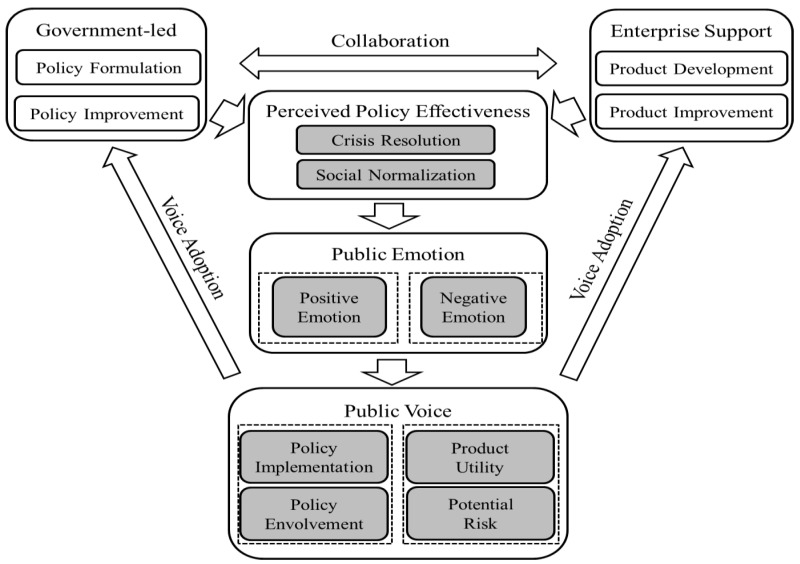
The model of public voice’s formulation and effects. Main categories are shaded grey.

On the basis of axial coding, selective coding excavates the core category from main categories and analyzes the connection relationship among them. As shown in Figure 1, the dynamic mechanism of public voice behavior to promote policy implementation and evolution in public health emergencies is as follows: First, under the guidance of the government, enterprises participate in the development of policy and design products to assist policy implementation with advanced technologies. Second, in response to the government policy, the public will use enterprise products in their daily life and work. And through judging whether the policy can effectively solve the current problems and guide the future development of the society to form the policy effectiveness perception. Third, public’s perception of the effectiveness of policies will trigger public emotions. Different perceptions of policy effectiveness can lead to positive or negative emotions. Then, emotions can induce public voice behavior, including voice for government policies and for enterprise products. Finally, the government and enterprises will give feedback to the public voice and improve the policies and products accordingly. As a new external stimulus, the improved policies and products also have an impact on the public’s perception of policy effectiveness, forming a dynamic mechanism of public suggestions to promote policy evolution and product innovation, as shown in Figure 1.

## 4. Results

Based on the results of grounded theory and cognitive appraisal theory of emotion, this paper constructs a driving mechanism of public voice behavior: “stimulus-cognition-emotion-behavior” model. The model shows that there are causal relationships among cognition, emotion, and behavior. According to the cognitive appraisal theory of emotion, under the stimulation of external events, the external information obtained by individuals first enters the perceptual system for compilation and processing, forming specific cognitions. Cognitions trigger the individual’s emotional response, and finally produces specific behavioral tendency [59].

### 4.1. The Formation Process of Public Voice (Stimulus-Cognition-Emotion-Behavior)

#### 4.1.1. Policy as the Stimulus

Public policy is the political and technical approach to solve problems, fundamentally, it is pragmatic [60]. Under the cooperative governance, the government is no longer the only decision-maker, but the main participant plays a guiding role [49]. With the advent of the new Internet era, the impact of big data, cloud computing, and other technologies on policy formulation and implementation cannot be ignored. First of all, the Internet can optimize the link of policy-making, and the process of it can be completed with the help of the Internet, thus making policy-making more efficient. Secondly, big data can provide a wider range of data sources for policy evolution. Through data mining and analysis, it can provide big data support for policy evolution, making policy formulation and implementation more reasonable. Finally, the open data system can further broaden the channels for the public to participate in policy discussions and make policy-making more democratic. Due to the immature application of big data by the government and lack of professional talents, enterprises are required to provide technical support. The technical support of enterprises is more important for the formulation of policies under public health emergencies. As public health emergencies tend to be urgent, destructive, and uncertain, putting forward higher requirements for the timeliness, scientificity, and effectiveness of policies. In this case, it is very necessary for the government to cooperate with enterprises to formulate policies. The government is responsible for policy formulation and implementation, while enterprises take technological advantages to provide products or services to assist policy implementation.

#### 4.1.2. External Stimulus Leads to Perceived Policy Effectiveness

According to the cognitive appraisal theory of emotion, when individuals encounter the external stimuli, they will experience two-stage cognitive appraisal processes: primary appraisal and secondary appraisal. In addition, through the appraisal, people can assess the relevance of external stimuli to themselves and whether the resources they have can cope with the situation [61]. In public health emergencies, the policy launched by government-enterprise cooperation is an external stimulus for the public. Additionally, public appraisal mainly focuses on whether the policy can achieve policy purpose and effectively solve specific public problems, that is, perceived policy effectiveness. Under the policy stimulation, the public will use the cognitive system to make evolution of it [62]. The perception of policy effectiveness reflects the individual’s judgment of the correlation between the policy and himself and is an important way for policy to act on public behavior. A high level of perceived policy effectiveness indicates that the public believes the policy is beneficial to their daily life, while on the contrary, they consider that the policy has no significant positive impact or may pose a threat. Policy is the action route or method to guide the current and future decision-making, and its role should not be limited to solving the current problems, but also should be instructive for future development of society [63]. According to the results of analysis, the policy effectiveness in public health emergency includes crisis resolution and social normalization. In the case of public health emergencies, the first problem to be solved by policies is to reduce the adverse impact of emergencies, that is crisis resolution. On the premise that the crisis is under control, policies should also have effects of accelerating the social normalization and promoting economic recovery, that is, the social normalization function. Taking the Health Code policy as an example, if the public thinks that the Health Code policy cannot effectively control the spread of COVID-19, or cannot speed up work resumption, the public’s perceived effectiveness of Health Code policy will be low. Otherwise, the perception will be high.

#### 4.1.3. Perceived Policy Effectiveness Arouses Public Emotion

Emotions are the products of an individual’s appraisal of the person–environment relationship and of great diagnostic value to help an individual identify what is important under a specific situation. Additionally, emotions vary with the change of appraisals [61]. The public’s emotional response to policy is formed on the basis of perceived policy effectiveness. According to the cognitive appraisal theory of emotion, emotion intuitively shows the public’s evolution of external stimulus perception, and its core is evaluative cognition. Almost everything will stimulate people to produce emotion, no matter if it happens or not [62]. However, emotion cannot be aroused by external stimulus directly; the appraisal process of relationship between person–environment is necessary to evoke emotion. When individuals are in a certain situation, they will evaluate it, be satisfied or dissatisfied, beneficial or harmful, and make corresponding emotional reactions [59]. If perceived policy effectiveness is high, the public will have a positive emotion, or vice versa. Taking the Health Code policy as an example, different perceptions of public policy effectiveness will stimulate different emotions. When the public perceive that the Health Code policy can effectively control the spread of COVID-19 or accelerate economic recovery, they will generate positive emotions. Otherwise, they will hold negative emotions. Examples of comments about Health Code policy are as follows.

Comment 1: As I am from Hubei province, I didn’t go back to my hometown, so I couldn’t enter the market for 20 days. After having the health code, I entered the market for the first time without being stopped. It is easy to use and it’s really convenient, give it a thumb up!

Comment 2: I’m in Fuyang, and I’m not even allowed to go to my husband’s hometown in the countryside. I haven’t left Fuyang for nearly a month. I haven’t even gone to downtown or move around Fuyang. What the hell is this code? I don’t understand. I’m so angry!

#### 4.1.4. Public Emotion Stimulates Public Voice

According to the cognitive appraisal theory of emotion, the cognition and appraisal of external environment will stimulate special emotions. Then, the emotion will motivate coping behaviors to prevent harm or to improve the prospects for benefit [64]. Public voice behavior is generated under the influence of public emotions. According to cognitive appraisal theory of emotion, emotional response will lead to an individual’s specific behavior tendency to regulate the emotion (emotion-focused coping) or change for the better the problem (problem-focused coping) [61]. On the basis of the public perceived policy effectiveness, the emotional reaction is finally transformed into the driving force to improve the effectiveness of the policy, which urges the public to put forward a constructive voice or point out the problems existing in the policies and products. When the public believe the policy can effectively defuse the current crisis and benefit future development, they will hold positive emotions and employ behaviors that can maximize the policy benefits. However, when the public think that there are some defects in the process of policy implementation undermining the policy effectiveness, they will generate negative emotions and take actions to reduce potential harm. After analysis, it is found that public voice can be divided into two dimensions: policy voice and product voice. According to the content of voice, policy voice can be divided into policy evolution and policy implementation. Policy evolution voice is promotive voice and usually occurs when the public is in positive emotion, referring to the public’s suggestions on the promotion and unification of policies across the nation. Policy implementation voice refers to the voice made by the public for the actual implementation process of policies. In a public health emergency, policy implementation voice is mainly in the form of pointing out defects in the process of policy implementation, and it usually happens when the public is in negative emotion. Public voice on products can be divided into product utility and potential risk. Product utility voice refers to the public’s suggestions on improving product efficiency and it includes both promotive voice as well as prohibitive voice. While potential risk voice is prohibitive voice, referring to the public’s concern about the negative effects caused by enterprise’s products. The examples of the Health Code policy are shown below.

Comment 3: Now in many provinces, the biggest problem is that people are not allowed to enter the community! Not even people with health codes! This is too unreasonable! If a policy is made, it is to be implemented. What good is policy if the implementation problem at the grassroots level is not solved?

Comment 4: The Health Code really gives me a great convenience in my life. It’s easy to go out with it. I hope it can be promoted nationwide.

In conclusion, the formation process of public voice behavior conforms to the “stimulus-cognition-emotion-behavior” model of cognitive appraisal theory of emotion. Policy stimulus leads to the public’s cognition of the effectiveness of policy, which arouses public emotion response and further leads to public voice behavior.

### 4.2. The Dynamic Process of Public Voice on Policy Evolution

**Figure 2:**
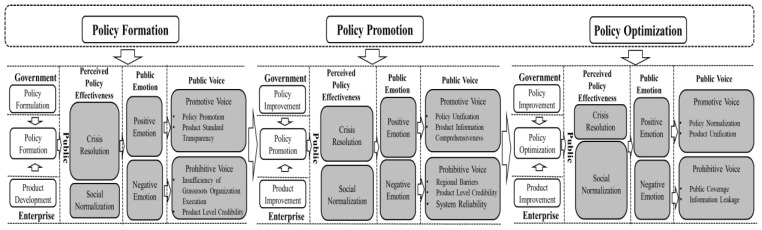
Model of the dynamic effect of public voice behavior on policy, results of grounded theory are shaded grey.

The formation and evolution of policy is a dynamic and continuous process. Previous studies have paid more attention to the impact of public participation in the policy-making stage [65,66]. However, this study finds that after policies are made, public voice also has a great impact on the evolution and implementation of policies. Based on the results of grounded analysis, this paper divides the process of policy evolution into three stages: policy formation, policy promotion, and policy optimization, and constructs a dynamic mechanism of public voice to promote policy evolution and product innovation, as shown in Figure 2.

#### 4.2.1. Policy Formation Stage

The policy is formed in accordance with the rigorous policy-making process in order to solve specific public problems. As the output of the political system, the main function of policy is to solve social public problems effectively. As public health emergencies often pose a major threat to social security and public order, as well as the safety of citizens’ lives and property, the policy under public health emergencies aims to resolve the crisis state timely and effectively and restore the normal life order as soon as possible [15]. As an external stimulus, the formation of policies will lead to the public’s perceived policy effectiveness. At this stage, citizens’ cognition of policy effectiveness mainly focuses on crisis resolution. Whether the policy can effectively alleviate the adverse impact of public health emergencies is an important factor affecting public emotion. When policy is implemented, the public will form the perception of whether the policy can resolve the crisis effectively. When perceived policy effectiveness is high, the public will have positive emotion and tend to conduct promotive voice. As the construction of national emergency management system follows the basic principles of “ability-standard” and “center of gravity down”, the local government is in the front line when dealing with public health emergencies and bears the main responsibility. Therefore, the policies under public health emergencies are often formulated by the local government, and the superior government selectively promotes the policies according to the evolution of the applicability. So the public will suggest to promote policy across the country if they think the policy is effective enough. Besides, the public will provide promotive voice to improve product utility in a state of positive emotion. When the public perceive the policy is not effective enough, they will have negative emotion and tend to conduct a prohibitive voice. Public health emergencies prevent policy-making from following a strictly procedural process. The government needs to complete the implementation of the policy in a limited time, and it is difficult to guarantee the implementation of the grassroots administrative staff in a short time [15]. Therefore, the prohibitive voice mainly focuses on pointing out the problems existing in the implementation of the policy at the grassroots level. In the policy formation stage, as the implementation of enterprise’s product auxiliary policy, the public’s requirements for its effectiveness are more stringent. Therefore, the public will be more active in pointing out problems in the use of products.

#### 4.2.2. Policy Promotion Stage

Through the evolution and adoption of public voice, the government improves the policy and policy evolution enters the policy promotion stage. As a new external stimulus, the improved policy continues to act on public cognition. More than that, the focus of perceived policy effectiveness begins to shift from crisis resolution to social normalization. Under the control of the government, the grassroots implementation has been further improved, and the effectiveness of policies to solve current problems (i.e., crisis resolution effectiveness) has been effectively played. However, the effects of policies cannot limit to provide methods to solve the current problems, but also play a guiding role in the future development of society [63]. Public health emergencies make society change from normal state to emergency state, which has a great impact on public life and work [15]. Therefore, on the basis of effective resolution of the crisis, whether the policy can further promote the recovery of social normality has been widely concerned by the public. Under the influence of public emotion caused by the cognition of policy utility, voice behavior emerges. In the stage of policy promotion, public promotive voice is policy unification. Government policy-making under public health emergencies emphasizes the local government’s ability of ‘territorial management’. However, with the promotion of local policies across the country, the problem of compatibility between policies begins to emerge. The inconsistency of policies in different regions will bring many inconveniences to the public. Therefore, in order to improve the effectiveness of policies, the public suggests that policies should be unified across the country. At this stage, with policy promotion, the audience range of the product is constantly expanding, the public’s attention to the product utility is also increased. Improvement suggestions to enhance the effectiveness are still the focus of voice. However, in addition to the utility of the product, the public also began to pay attention to the use experience of the product, pointing out the problems of the system in the use process.

#### 4.2.3. Policy Optimization Stage

Under the influence of public voice, the government and enterprises constantly improve the policies and products, and the policy evolution enters the optimization stage. At this time, with the public health emergency in the rehabilitation stage, the effectiveness of the policy has been played out to a greater extent; the public urgently need to return to normal life and work state, so the focus of perceived policy effectiveness is social normalization. Public voice is still affected by the emotional response based on cognition, and the content of public voice has changed further. Considering the adverse impact of public health emergencies, with the purpose of preventing the recurrence of the public health emergency, the public suggest that the policy should be normalized. Policy normalization can predict the occurrence of public health emergencies in the early stage and minimize the loss. In addition, the public begin to pay attention to the coverage of the policy, pointing out that the omission of the population covered by the policy may have a negative impact on the fairness. With regards to the products of enterprises, the public voice focuses on the risks of long-term use of products. Products are tools for enterprises to participate in cooperative governance and used to supply policy implementation. With the help of products, enterprises take part of the responsibilities originally belonging to the government, which will cause public concern.

To sum up, public voice plays an important role in the evolution of policies. First, public opinion provides the widest source of information for policy feedback. Public health emergencies require the government to formulate effective policies in the shortest time based on the least information and resources, and the effectiveness of the policies is uncertain [15]. The public voice gives quick feedback to the policy, which provides the basis for the government to evaluate the effectiveness of the policy. Second, the public voice expresses the public interest demands and promotes the policy to be more democratic and efficient [67]. In order to gain more and more public support in the process of policy-making, public voice is an important consideration for the government in the process of formulating and implementing policies. Finally, public voice behavior also plays an important role in product improvement and innovation. It can be seen from the analysis, that in the policy of government enterprise cooperation, due to the particularity of the product, the public’s requirements are more stringent. Voice for product improvement aims at making it more suitable to assist policy implementation, and it will provide an important reference for enterprise product innovation.

## 5. Conclusions

This study reveals the driving mechanism of public voice behavior and enriches the literature on voice behavior. First, based on the results of qualitative research, this paper employs the cognitive appraisal theory of emotion to explain the process of formation of public voice behavior under public health emergencies, via the stimulus-cognition-emotion-behavior model. Unlike voice within an organization, public voice on social media is a kind of self-motivated behavior free from the pressure of peers and organizational climate [4]. What is more, as the purpose of public voice is to improve social status quo, the cognitive appraisal theory of emotion is eminently suitable for explaining the formation of public voice behavior. As an external stimulus, a policy will have an impact on the public’s cognitive processes, and prompt them to evaluate whether the policy can resolve a current issue and play a guiding role in the future development of society. When the public perceives the policy to be highly effective, they will have positive emotions; otherwise, they will have negative emotions. Take the Health Code policy as an example, if the public think that the policy can effectively contain the spread of the novel coronavirus and speed up the resumption of the normal activities, they will experience positive emotions, and vice versa. In accordance with the cognitive appraisal theory of emotion, emotional response will stimulate behavioral tendencies. The public’s positive emotions will lead them to employ a promotive voice to expand the effectiveness and coverage of the policy, whereas the public has the tendency to use prohibitive voice to reduce the possible negative effects of a policy when they are not satisfied with its effectiveness. This result is consistent with previous studies that make a clear distinction between promotive voice and prohibitive voice, where the former is positive in tone and the later negative [68]. In this study, members of the public feeling positive emotions will voice to promote a policy and establish uniform standards throughout the nation, whereas those experiencing negative emotions will identify deficiencies such as implementation at the grassroots level. Second, this study clarifies the objects and types of public voice. Compared with employee voice and customer voice, the coverage of public voice is more extensive. Thus, for different problems, the objects of public voice are also different, which require separate analysis in each situation. Under this circumstance, the objects of public voice include two main bodies involved in policy-making: governments and enterprises. For the Health Code policy, the objects of public voice are the government, Alibaba, and Tencent. With regard to voice type, there is some similarity with the other two kinds of voice—public voice can also be divided into promotive voice and prohibitive voice. Finally, through qualitative research, this paper has attempted to reveal the role of public voice in policy evolution and product innovation, clarifying the promoting effect of public voice on societal improvement. The study emphasizes the importance of public voice via social media, suggesting that both government and enterprises ought to attach more significance to public voice when making decisions.

Taking China’s Health Code policy under COVID-19 as an example, this paper has constructed a dynamic mechanism for the effects of public voice on policy evolution. The study focused on the promotion of public voice for policy improvement and evolution in the late stages of policy-making. Public opinion contains information about demands and aspirations which is very valuable for decision makers. To absorb more public opinions and take into account public aspirations or priorities before policy formulation, previous research has paid much attention to the impact of public opinions at the pre-policy-making stage [65,66]. No studies have examined the impact of public voice on policy after its implementation. The development of social media not only provides a wider source of information for the public, but also builds a more convenient platform for the public to voice their opinions at any stage of policy formulation or implementation, thus having effect on policy. This study shows that after a policy is implemented, public voice is still of great value for policy evolution. However, this study divides policy evolution into three stages: policy formation, policy promotion, and policy optimization. It introduces changes in public policy utility perception and public voice content at different stages, and constructs the dynamic mechanism of the effect of public advice on policy improvement based on the government’s adoption of public advice to promote policy evolution and implementation.

To some extent, this study provides support for cooperative governance research. Cooperative governance has different connotations in different situations, and there are also some differences among participants. The formulation and evolution of policies under public health emergencies is an important practice of cooperative governance. Faced with a public health emergency, the government, enterprises, and citizens should form an open overall system to jointly govern social public affairs. The government, enterprises, and individuals play their own roles, participate and cooperate with each other to effectively reduce the negative impacts of a crisis and maintain the stable development of society. In this process, governments, enterprises, and the public are in a more equal position, and multi-agent participation is truly realized. Faced with COVID-19, Yaowen Wang, deputy director of Shenzhen Municipal Government Service Data Management Bureau, said the epidemic situation was a great challenge to the government’s governance ability and level. In addition, the fundamental problem was laid in whether the whole society could be quickly mobilized and organized to participate in the prevention and control in a short period of time. As an organ of power, the government is responsible for the formulation and implementation of policies. Enterprises participate in the formulation of policies, and provide products and services with technical advantages to assist with policy implementation. As for the public, in addition to regulating their own behaviors under the guidance of policies, they also provide feedback and voice on policies and enterprises’ products and services. Take China’s Health Code policy as an example, the government is responsible for the formulation and implementation of the policy. Alibaba and Tencent are committed to the development and updating of the health code system and participate in the formulation of the policy standards. The public need to move around in strict accordance with the policy guidelines and actively provide voice. Under a public health emergency, public voice is an important way for public to participate in cooperative governance. It provides real-time feedback for policy, helping government and enterprises to make decisions as quickly as possible and set aside more time to fight against emergencies. Further, public voice can facilitate the promotion of effective policy, improving prevention efficiency. As a universal way of participating in cooperative governance, public voice via social media deserves more attention in the future. 

Although this research makes several contributions, there are still some limitations. First, we studied the influence of public voice only on policy evolution and specific product innovation. As public voice is social-oriented, it will affect almost all social affairs and phenomena. Future research can explore the influence of public voice behavior in other respects. Second, this study revealed the generative mechanism of public voice behavior from the perspective of emotional cognition. As a self-oriented behavior, public voice may be triggered by other internal processes. Future research could explore the antecedents of public voice from different perspectives. Third, this study was conducted under a public health emergency, COVID-19. As public emergencies take several forms, the results differ in different situations. Future research might examine public voice in other contexts. Fourth, although many countries and regions have formulated corresponding policies in the context of public health emergencies, results from the study of China’s Health Code policy under COVID-19 may not be fully applicable to other nations, and future research should be conducted in different cultural contexts.

## Figures and Tables

**Table 1:** Examples of open coding.

Category	Concept	Example from Original Text
System Reliability	Filling mechanism	The above is all my own writing There will be human factors to fill in the health code all by myself
	Feedback mechanism	I never get in when call customer service consultation I didn’t get any answer when I called 12345, and problems have not been dealt with after I reported to Alipay
	System fluency	Sometimes I cannot get in the system Yesterday the system crashed and it provided green code only
	Clock mechanism	Now the code is yellow. It says that the seven-day clock normally changes to green code, but I haven’t been able to clock in for three days in a row. Is it a dead cycle I couldn’t clock in
	Quantitative limitation	I applied early this morning and being told there were no place available today There is a quantity limit on health code, I can’t apply for it
	Technical defects	There’s a problem with the back-end technology
Ensure resumption of work and production	Risk control	This kind of formalism will only make Hangzhou more dangerous. Some areas do not need to be quarantined if they have proof of returning to work. Is such a perfunctory anti-epidemic measure really safe? Replacing current containment measures with health codes has serious consequences
	Daily traffic	A few days ago, Shanghai swept this health code in highway traffic, it was very fast It’s really convenient to go out this way, and you don’t have to worry about losing the paper material
	Quarantine	The problem is that I came back on the 2nd. The quarantine for 14 days does not count as before. It means I will be quarantined for one month Recently, cross provincial commuting is becoming crazy. There are 14 days of isolation at work and 14 days after work
	Checking routine	It’s much more convenient than running around to apply material This is not only efficient to reduce the burden of screening personnel, but also can record personal travel

**Table 2:** Axial coding and analysis results.

Classification	Main Category	Category	Connotation
Perceived policy effectiveness	Crisis resolution	Ensure personal safety	Reduce the impact of public health emergencies on the public’s personal and property safety
		Reduce public burden	Reduce the burden of public health emergencies on the public’s daily life and psychology
	Social normalization	Facilitate personnel flow	Perception of the effectiveness of policy in facilitating mobility
		Ensure resumption of work and production	Perceived effectiveness of the policy in ensuring the safety and improving the efficiency of the resumption of work and production
Public emotion		Promote economic recovery	Perceived effectiveness of policy in getting society back on track and restoring the economy
	Positive emotion	Satisfaction	Emotion arises when perceived policy effectiveness lives up to expectation
	Negative emotion	Dissatisfaction	Emotion arises when perceived policy effectiveness falls short of expectation
Public voice	Policy evolution	Policy promotion	Voice proposed by the public to promote local policies nationwide
		Policy unification	Voice proposed by the public to unify local policies
		Policy normalization	Voice proposed by the public to carry out the policy under normal circumstances
	Policy implementation	Execution of grassroots organization	Implementation of government policies by grassroots workers
		Regional barriers	The problem of poor policy compatibility caused by different local policies
		User coverage	The policy is inadequate in terms of population coverage
	Product utility	System reliability	Reliability of the product system itself
		Level credibility	How reliable is the product to the user’s health rating
	Potential risk	Information disclosure	There is a risk of information leakage when the product collects too much user information
		Abuse of power	There is a risk of abuse of rights when enterprises assume part of government responsibilities
